# Physical Stability and Antioxidant Ability of a Sustainable Oil-in-Water Emulsion Containing Perilla Skin Extract and Upcycled Aquasoya Powder

**DOI:** 10.3390/foods13071063

**Published:** 2024-03-29

**Authors:** Bo-Kyong Kang, Jing-Chao Yu, Weon-Sun Shin

**Affiliations:** Department of Food and Nutrition, College of Human Ecology, Hanyang University, 17 Haengdang-dong, Seongdong-gu, Seoul 04763, Republic of Korea; rkdqhrud326@gmail.com (B.-K.K.); yujingchao@hanyang.ac.kr (J.-C.Y.)

**Keywords:** upcycled aquasoya, rosmarinic acid, encapsulation efficiency, functional emulsion, antioxidant activity, food application

## Abstract

In response to environmental issues, upcycling has become a growing trend in the food industry. Aquasoya is a promising method to upcycle by-product from soybean processing due to its high protein contents and excellent emulsifying ability. In the present research, Aquasoya powder was used an emulsifier to incorporate the antioxidant compounds from perilla skin extract (PSE), namely rosmarinic acid, into oil-in-water (O/W) emulsion system and its physochemical stability was assessed. As a result, droplet size of the emulsion was smaller in PSE-incorporated emulsion (PO, 350.57 ± 9.60 ^b^ nm) than the emulsion without PSE (PX, 1045.37 ± 142.63 ^a^ nm). Centrifugal photosedimentometry analysis also revealed that the physical stability was significantly improved in PO, and the stability was maintained over 30 d of storage. Furthermore, as PO had a higher ABTS radical scavenging ability and showed slower initial lipid oxidation, it was concluded that PO has a higher antioxidant ability than PX. Conclusively, Aquasoya can be considered as an emulsifier in O/W emulsion with PSE because it can effectively integrate and stabilize the antioxidant substance derived from perilla skin.

## 1. Introduction

Growing concerns regarding the environmental issues have prompted food scientists and researchers to identify sustainable ingredients for food production. The overall food system accounts for one-third of global greenhouse gas emissions, and food waste accounts for approximately 8–10% of all human-caused emissions [1]. A new concept, known as “food upcycling”, has been introduced to reduce gas emission from food waste and alleviate this problem. Upcycling refers to the new identification of by-products. Food products can be classified as upcycled foods when they meet three criteria: (1) they contain materials that would otherwise be discarded if not recycled, (2) they are converted into food products suitable for human consumption, and (3) their production involves a process that adds value to the materials [2]. Such products are expected to have crucial potential for sustainable transitions in agricultural and food sectors.

Legumes are a good source of carbohydrates, proteins, dietary fibers, lipids, minerals, and vitamins. Soaking and boiling are required to transform the dried legumes into food and food products. However, these processes result in large volumes of liquid by-products that are rich in nutrients. Aquasoya is a promising method for recovering proteins from discarded materials. It is defined as the thick liquid produced when soybeans are boiled in water. Echeverria-Jaramillo et al. compared the nutritional contents and emulsifying/foaming properties of Aquasoya from three different Korean legumes, namely, black soybeans, yellow soybeans, and small black beans (SBB), and aquafaba, the cooking water from chickpea [3]. The authors found that Aquasoya had higher total polyphenol, total carbohydrate, and protein contents, as well as better emulsifying and foaming abilities than aquafaba. Therefore, recent studies have identified ways to apply it to vegan bakery products and further improve their functionality [4,5,6], verifying its potential use in food products as an upcycled ingredient. However, their applicability as an emulsifier into bioactive compounds-formulated oil-in-water (O/W) emulsion systems has yet been assessed. For this reason, relevant studies are required to expand the use of Aquasoya in food production, thus attributing to achieve environmental protection through upcycling of social resources.

*Perilla frutescens* (L.) Britt is an annual herbal plant belonging to the Lamiaceae family. Owning to its various health benefits, it is widely used in edible and medicinal applications in many Asian countries, including Republic of Korea, China, and Japan. As perilla seed oil is rich in polyunsaturated fatty acids such as linolenic acid, linoleic acid, and oleic acid, it can prevent age-related cognition problems and mental decline [7]. Perilla seeds also show diverse physiological effects such as antioxidant and antibacterial effects. Yamamoto and Ogawa found that perilla seed extract can work as antimicrobial agent that could prevent dental caries and periodontal diseases [8]; an important factor contributing to these physiological effects is high contents of phenolic compounds, namely, rosmarinic acid (RA) and its derivatives.

RA is an ester of caffeic acid and 3,4-dihydroxyphenyllactic acid, which are commonly found in Boraginaceae and the Nepetoideae subfamily of Lamiaceae (rosemary, lemon balm, marjoram, oregano, etc.) [9]. RA has various biological properties, including antioxidant, antibacterial, anti-inflammatory, and antiviral activities. Lee et al. identified the phenolic compound profiles and antioxidant properties of the seeds of various perilla cultivars and investigated the relationship between total polyphenolic content and the antioxidant ability of each cultivar. Nine compounds were identified, among which RA and RA-3-O-glucosidase showed the highest contents (>95%); moreover, the antioxidant effects of perilla seeds were correlated with their phenolic content [10].

Perilla seeds are frequently consumed in the form of oil and powder rather than its native form. After processing, perilla skin and perilla pomace are usually considered waste and discarded. However, as these by-products have high nutritional value and contain phenolic compounds, carbohydrates, and proteins, various attempts have been made to develop methods to upcycle them [11,12,13,14]. Chumphukam et al. also found that perilla seeds and perilla seed meal extracts exert potential anti-mutagenic, antioxidant, and anti-inflammatory effects, and RA was predominantly found in both extracts [15]. Nevertheless, perilla by-product extracts’ practical application in food systems has not been widely investigated. Besides, RA shows an intrinsic low lipophilic nature, and the stability of RA can be impaired due to the external stress such as the acidic environment and enzymes in many food products [16]. For this reason, strategic approaches are required to successfully corporate RA from perilla by-products into a food system.

**Figure 1:**
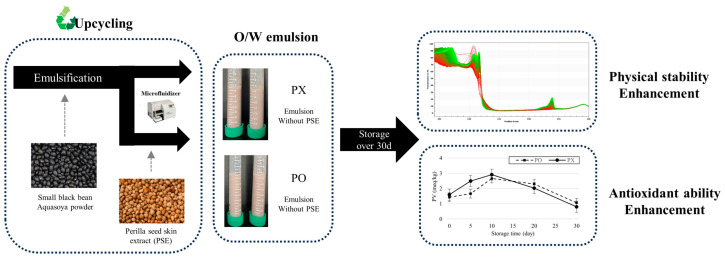
Schematic diagram of depicting the workflow and results of this research.

In this study, a sustainable O/W emulsion containing perilla skin extract (PSE) and perilla oil was formulated with upcycled SBB Aquasoya powder, and its physicochemical properties were evaluated. Schematic diagram of this research is shown in Figure 1. PSE was used as a source of antioxidant compounds including RA and protein-recovered upcycled SBB Aquasoya powder (rSBB) functioned as an emulsifier. As RA is a phenolic compound which can potentially interact with emulsion droplets, particle size, ζ-potential, and centrifugal photosedimentometry analyses were performed to determine whether PSE influenced physical stability of the emulsion. Furthermore, perilla oil is susceptible to lipid oxidation due to its high polyunsaturated fatty acid contents, 2,2-diphenyl-1-picryhydrazyl (DPPH), 2,2′-azino-bis(3-ethylbenzothoazolin-6-sulfonic acid) diammonium salt (ABTS), and peroxide value (PV) assays were performed to determine whether the bioactive compounds from PSE could still function as antioxidant reagents after incorporation into the emulsion system.

## 2. Materials and Methods

### 2.1. Plant Material and Chemical Reagents

The perilla cultivar Nulsaemi (*Perilla frutescens* (L.) Britton var. frutescens) was obtained from a farm in Gangwon-do, South Korea (38°03′05.9″ N 127°48′59.4″ E), and a bottle of virgin perilla oil (150 mL), made solely from raw perilla seeds, was purchased from a local grocery store “Hansallim” in Seoul, Republic of Korea. n-Hexane, ethanol, anhydrous monobasic sodium phosphate and acetic acid were acquired from Samchun Chemical Co. (Seoul, Republic of Korea). DPPH and ABTS were purchased from Sigma-Aldrich Co. (St. Louis, MO, USA). Chloroform, potassium iodide, sodium thiosulfate, and soluble starch were purchased from Daejung Chemicals and Metals Co. (Daejeon, Republic of Korea). All these chemical reagents were of analytical grade. High-performance liquid chromatography (HPLC)-grade acetonitrile was obtained from Burdick & Jackson (Muskegan, MI, USA), and HPLC water was acquired from J. T Baker (Phillipsburg, NJ, USA).

### 2.2. Protein Recovery from SBB Aquasoya Powder

Protein recovery from SBB Aquasoya powder was performed by following the method described by Kim and Shin, with slight modifications [5]. The spray-dried SBB powder was dissolved in distilled water (DW) at a concentration of 100 mg/mL to make 500 mL of SBB solution, and the pH was adjusted to 4.5 using 1 M hydrogen chloride (HCl). The precipitated solution was centrifuged at 3915× *g* for 15 min, and the sediment was washed three times with 350 mL of DW. The precipitate was dispersed in 300 mL of DW, the solution pH was adjusted to 7 with 1M sodium hydroxide (NaOH), and the resulting suspension was lyophilized. The dried cube was ground with a pestle and mortar and processed using a 200 μm sieve.

### 2.3. Phenolic Compound Extraction from Perilla Skin and Powder

Extraction of phenolic compounds from perilla skin and perilla powder were conducted according to the method described by Guan et al., with slight modifications [17]. The skin was removed from the perilla seeds and then the remnants were milled to produce perilla powder. The perilla skin and perilla powder were then ground using a mixer and kept in a drying oven at 45 °C overnight. Because pomace means solid remnants after pressing for juice or oil, we extracted 4 g of the pulverized perilla skin in a Soxhlet extractor using n-hexane (80 mL) at 71 °C for 4 h to remove crude oil constituents and form perilla pomace from perilla. This procedure was also performed using perilla powder for consistency. The resulting residue was stored at 71 °C to evaporate the remaining solvent. Phenolic compounds from the remnants were extracted with 80% ethanol at 82 °C for 6 h. The infusion was passed through a Whatman No. 1 filter paper, and the solvent was removed using a rotary evaporator at 50 °C. The residual substances were detached from the flask by dissolution in DW, after which they were lyophilized and stored at −20 °C for further analysis.

### 2.4. HPLC Conditions for RA Quantification

HPLC was performed as described by Hong and Kim [18]. RA constituents in perilla skin and perilla powder were identified using an Agilent HP1200 series instrument with a SunFire^®^ C18 column (5 μm, 4.6 × 250 mm). Each extract sample (i.e., perilla skin extract (PSE) and perilla powder extract (PDE)) was dissolved in 80% ethanol. The injection volume was 20 μL. Isocratic elution was performed using acetonitrile–water (22:78 *v*/*v*) containing 1% acetic acid as the mobile phase. The flow rate was set to 1 mL/min, and each compound was detected at 320 nm. The RA content in the samples was determined by comparing their integrated peak areas with a standard graph. The standard graph was plotted by diluting RA stock standard solutions (concentration range, 20–100 μg/mL) with 80% ethanol. The sample and standard solutions were filtered through 0.20 μm polytetrafluoroethylene syringe filter prior to HPLC analysis.

### 2.5. Encapsulation Efficiency of RA

The encapsulation efficiency of RA was determined according to the method described by Arranz et al. [19]. rSBB was dissolved in 50 mM sodium phosphate buffer (SPB, pH 7.4) (100 mg/mL). Subsequently, 0.2 mL of the rSBB solution was mixed with 5 mg/mL ethanolic PSE stock solution and a solvent (ethanol:50 mM SPB (pH 7.4) (1:3 *v*/*v*)) to obtain 0.1, 0.2, 0.4, 0.8, and 1 mg/mL PSE. Each mixture (1.2 mL) was maintained for 14 h with stirring. These samples were centrifuged using an M13 series instrument (Hanil Scientific Inc., Gimpo, Republic of Korea) for 15 min at 3000× *g*. Then, 10 μL of the supernatant was collected and dissolved in 80% ethanol. RA contents in supernatant was quantified by HPLC analysis using the method described in Section 2.4 and calculated as follows:

$$Encapsulation\;Efficiency\;(\%)=\frac{R_{s}}{R_{t}}\times 100$$

where R_t_ is the theoretical RA content in the RA-rSBB mixture and R_s_ is the amount of RA in the supernatant determined by HPLC quantification.

### 2.6. Preparation of Emulsion Samples

The O/W emulsions were prepared by the method described by Han et al., with slight modifications [20]. Perilla oil (10.5 mL) was dispersed in rSBB or rSBB-PSE solution. Perilla oil (5%, 10.5 mL) was added to 35 mL of the rSBB-SPB solution (100 mg/mL). The emulsion sample with PSE (PO) was prepared with 16.8 mL of ethanolic PSE stock solution (5 mg/mL), while that without PSE (PX) was prepared using a solvent (ethanol:50 mM SPB (pH 7.4) (1:3 *v*/*v*)) to replace the same volume of PSE stock solution. The final volume was 210 mL. The mixtures were homogenized at 11,000 rpm for 1 min and at 11,200 rpm for 1.25 min using an Ultra-Turrax T25 disperser (IKA, Staufen, Germany). Subsequently high-pressure homogenization was then performed at 600 bar for one pass using a microfluidizer (LM20; Microfluidics, MA, USA). The emulsions were stored at 4 °C for 30 d to determine changes in their stability and antioxidant ability.

### 2.7. Emulsion Characteristics

#### 2.7.1. Droplet Size and ζ-Potential

The particle size and polydispersity index (PDI) of the emulsion samples were determined using a ZEN 1690 series instrument (Malvern Instruments, Malvern, UK). The emulsions were diluted 50 times in a solvent (ethanol: 50 mM SPB (pH 7.4) (1:3 *v*/*v*)) to avoid multiple scattering.

The electrical charge (ζ-potential) of the emulsion droplets was examined using a Zetasizer Pro instrument (Malvern Instruments, Malvern, UK). Prior to measuring ζ-potential, the emulsion samples were mixed homogenously with solvent to form the aqueous phase.

Each measurement was performed at 25 °C and 0, 5, 10, 20, and 30 d after formulation.

#### 2.7.2. Physical Stability

The physical stability of the emulsions was evaluated using the method described by Zhi et al., with slight modifications [21]. A LUMiSizer centrifugal analyzer (LUMGmbH, Berlin, Germany) induced a creaming phenomenon or droplet aggregation by exerting a centrifugal force (2082× *g*, 4 °C, 60 min) on the samples, to draw the transmittance-height profiles of emulsion samples. This measurement was conducted at the day of emulsion formulation.

### 2.8. Antioxidant Ability Analysis

#### 2.8.1. DPPH Radical Scavenging Ability

The DPPH radical scavenging ability of the emulsions was determined using the method described by Huang et al., with slight modifications [22]. Briefly, 0.5 mL of the emulsion samples was added to 0.5 mL of ethanolic DPPH solution (0.1 mM). The mixture was vortexed vigorously and incubated in the dark for 30 min at ambient temperature. The samples were centrifuged at 1980× *g* for 3 min using an HM 150 IV series instrument (Hanil Science Inc., Gimpo, Republic of Korea), and their absorbance (A_t_) was recorded at 517 nm using a UV–visible spectrophotometer (Genesys 10S; Thermo Fisher, Walthan, MA, USA). DPPH radical scavenging ability (%) was calculated using the following equation:

$$DPPH\;radical\;scavenging\;ability\left(\%\right)=\left(1-\frac{A_{t}-A_{b}}{A_{c}}\right)\times 100$$

where A_b_ is the absorbance of the solution in which DPPH–ethanol was replaced with ethanol and A_c_ is the absorbance of the mixed solution containing 0.5 mL of ethanolic DPPH solution and 0.5 mL ethanol.

#### 2.8.2. ABTS Radical Scavenging Ability

ABTS radical scavenging ability was assessed using the method described by Lee et al. [10]. First, 7 mM ethanolic ABTS solution (5 mL) was mixed with an equal volume of 2.45 mM potassium persulfate solution, and the mixture was kept in the dark for 12–16 h. Next, the ABTS stock solution was diluted with ethanol to adjust its absorbance at 734 nm to 0.70 (±0.02). An aliquot of each sample (0.1 mL) was added to 0.9 mL of the diluted ABTS solution and stored in the dark for 30 min. Finally, the A_t_ of the sample was measured at 734 nm using a UV-visible spectrophotometer. ABTS scavenging ability (%) was calculated as a percentage using the following formula:

$$ABTS\;radical\;scavenging\;ability\;(\%)=\left(1-\frac{A_{t}}{A_{c}}\right)\times 100$$

where A_c_ is the absorbance of the mixed solution in which the sample was replaced with ethanol.

#### 2.8.3. Peroxide Value

The PV of the emulsions were determined using the method of Homayoonfal et al. [23], with slight modifications. Thirty milliliters of an acetic–chloroform mixture (3:2) were added to an aliquot of the emulsion samples (1 g). A saturated potassium iodide solution (0.5 mL) was then added to the mixture, which was subsequently stirred at 250 rpm for 1 min and incubated in the dark for 10 min. Next, 30 mL of DW was added to the mixture, which was then vigorously stirred at 650 rpm. Starch solution (0.5 mL) was added to this solution (1% *w*/*v*), and titration with sodium thiosulfate was performed until the blue color of the solution disappeared. PV was expressed in milliequivalents of peroxide oxygen per kilogram of sample and calculated using the following formula:

$$PV\;(meq/kg)=\frac{V_{b}\cdot N\cdot 1000}{w}$$

where V_b_ is the volume of sodium thiosulfate applied, N is the normality of sodium thiosulfate, and w is the emulsion weight.

### 2.9. Statistical Analysis

Each experiment was conducted thrice unless otherwise specified. Statistical differences were evaluated using the Mann–Whitney test with *t*-test or Duncan’s test with one-way analysis of variance using SPSS software (IBM SPSS Statistics 27.0; IBM^®^, Seoul, Republic of Korea).

## 3. Results and Discussion

### 3.1. HPLC Analysis for RA Quantification in Perilla Skin and Powder

**Figure 2:**
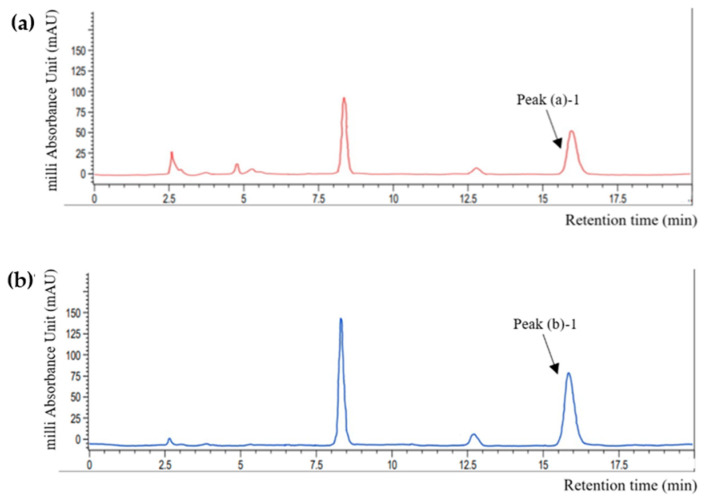
HPLC chromatograms of (**a**) perilla skin and (**b**) powder recorded at 320 nm. Peak (a)-2 and peak (b)-2 represents RA by comparison with HPLC results from RA standard solutions.

A comparative analysis was performed to assess the validity of perilla skin as a rosmarinic acid (RA) source. The high-performance liquid chromatography (HPLC) quantification results are shown in Figure 2 and Table 1. Comparison of the retention time of each peak with that of the standard RA solution (15.22 ± 0.71 ^N.S^ min) indicated that peak (a)-1 (15.75 ± 0.12 ^N.S^ min) and peak (b)-1 (15.608 ± 0.26 ^N.S^ min) represents RA (*p* > 0.05). There also were some other peaks that was higher in perilla powder than perilla skin. It has widely been explored that perilla seeds contains various phenolic compounds other than RA and its derivative, such as caffeic acid, luteolin and apigenin [10,15]. However, because this study more focuses on efficacy of perilla skin as a potential upcycling source of functional ingredients compared to perilla powder, and because their contents are commonly lower than RA and RA-O-glucoside, further identification of each peak was not performed.

Perilla skin exhibited an RA content lower than that of perilla powder. The RA contents in 1 g of dried perilla skin and perilla powder were 0.53 and 1.21 mg/g, respectively. According to a report from Korean Rural Development Administration, the RA content in Nulsaemi is approximately 2.13 mg/g [24]. The lower amount of extracted RA in this work may be attributed to differences in the extraction methods used. RA can be separated from herbal materials using appropriate extraction techniques, such as maceration, heat-reflux extraction (HRE), ultrasound-assisted extraction, and microwave-assisted extraction. The efficiency of these techniques was compared in previous research and found RA contents of 0.4–2.2 mg/g in rosemary [25]. The yield of HRE was higher than that of ultrasound-assisted extraction at the same temperature, but microwave-assisted extraction was the most suitable strategy for RA extraction. In the present research, HRE was applied due to its ease of operation and high yield rate. Temperature, extraction time, and solvent concentration can all influence the extraction yield [26]. The extraction was performed at 82 °C for 6 h with 80% ethanol as the extraction solvent. However, because RA is likely to be a heat-sensitive material, the extraction temperatures and time settings must be appropriate. Dent et al. investigated the extraction yield of phenolic compounds from Dalmatian Wild Sage (Salvia officinalis L.) using various solvents (30, 50, and 70% of acetone or ethanol) and temperatures (60 and 90 °C) [27]. Therefore, extracted phenolic contents, including RA, were lower at 90 °C, which could be attributed to phenolic compound degradation via hydrolysis, internal redox reactions, and polymerization. Meanwhile, Lee et al. used maceration method with 80% methanol and the extraction was conducted for seven days at room temperature. Subsequently, the contents of RA in eleven perilla cultivars were 1.08–2.52 mg/g [10]. Combining these preliminary research findings, it can be assumed that different extraction techniques and conditions resulted in lower extraction efficiencies. Because of its low toxicity, ethanol was used in this study for extraction, and the extraction temperature was slightly above the boiling point of ethanol. Therefore, more research is needed to determine the optimal extraction techniques and conditions that can lead to a higher extraction yield of phenolic compounds from perilla.

In addition, the RA content in the PSE was lower than that in the PDE. This result is not consistent with the results of previous studies. Guan et al. compared representative phenolic compounds (i.e., RA, RA-3-O-glucoside, luteolin, and apigenin) in the seeds and pomaces of 11 perilla cultivars and found that pomaces contained higher amounts of these substances than seeds [17]. The discordance between the results of the present study and the previous study could have originated from differences in the pomace formation procedures employed. While the skin was separated from the perilla seeds in this study, Guan et al. used whole seeds as the primary ingredient to prepare the pomace. Thus, the phenolic compound-rich matrix from the seeds may have blended with the formed pomace, resulting in higher phenolic contents. However, perilla skin still contained 33% of RA contents from perilla powder, and PSE and PDE showed only a 10% difference of RA contents. Based on these results, PSE was used to formulate an O/W emulsion containing phenolic compounds from perilla skin.

### 3.2. Encapsulation Efficiency of RA in rSBB-PSE Mixture

Encapsulation efficiency was analyzed to maximize the content of encapsulated RA in the O/W emulsions and achieve protective effects and increased viability. Encapsulation refers to the packing of small particles (core materials) into wall materials. This technique is usually employed to protect bioactive compounds (e.g., polyphenols, micronutrients, antioxidants, enzyme, and nutraceuticals) from unfavorable environmental conditions. Because RA is poorly soluble in water (1 mg/L) and has a low partition coefficient (log *K*_ow_ = 1.82) [28], a suitable wall material is required to enhance its storage stability.

Arranz et al. entrapped RA from marjoram (*Origanum majorana*) in soy protein isolate (SPI) and casein micelles and compared their encapsulation efficiency [19]. To minimize the insoluble fraction of the protein matrices, the authors set the final protein concentration of each solution to 5 mg/mL. SPI showed a higher encapsulation efficiency (20–30%) than caseins, and the best encapsulation was observed at the lowest concentration of the marjoram extract (0.1 mg/mL). Based on this study, we conducted a preliminary experiment to determine the optimal concentration range of PSE. When 2 mg/mL PSE was incorporated into 16.67 mg/mL rSBB-SPB solution, phase separation was observed. Thus, the PSE concentration in the rSBB-PSE mixtures was adjusted to 0.1, 0.2, 0.4, 0.8, and 1.0 mg/mL.

**Figure 3:**
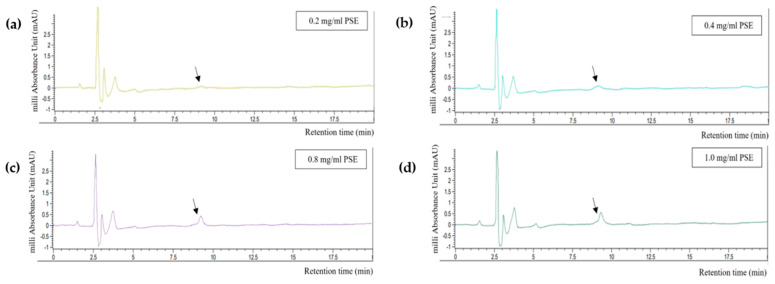
HPLC chromatograms of supernatants obtained from centrifugation of rSBB−RA mixture. (**a**) 0.2 mg/mL, (**b**) 0.4 mg/mL, (**c**) 0.8 mg/mL, and (**d**) 1 mg/mL of PSE.

The HPLC quantification results are shown in Figure 3. The encapsulation efficiency of RA was 100% at all concentrations tested because no peak appeared at 15 min, the time at which the peak of pure RA was detected during standard calibration. However, a peak appeared after approximately 8 min of retention (black arrows in Figure 3). This peak was also detected in the HPLC results of PSE and PDE, and its width was found to be proportional to the concentration of PSE. The peak became more distinct in the mixture with 0.8 mg/mL PSE. Thus, the concentration of PSE in the O/W emulsion was determined to be 0.4 mg/mL; this concentration ensured the optimal content of Aquasoya-protected RA in the formulation.

### 3.3. Emulsion Stability

Emulsions are colloidal dispersions of liquids embedded in different phases. Among emulsions, O/W emulsion has frequently been used for encapsulating bioactives such as antioxidants and vitamins that are lacking affinity for water [29]. As most emulsions are thermodynamically unstable, an emulsifier is usually added to prevent the emulsion from breaking. Proteins are frequently used as emulsifiers in O/W food emulsions because they are edible, amphiphilic, and water-soluble. They also provide remarkable resistance to coalescence [30]. Proteins can interact with polyphenols, forming either soluble or insoluble protein–polyphenol complexes. These structural changes can alter protein functionality [30]. Therefore, the physical characteristics of the emulsion samples were assessed to clarify whether PSE influenced the O/W emulsion system formulated using Aquasoya powder.

Table 2 lists the initial characteristics (mean droplet diameter, PDI, and ζ-potential) of the emulsion samples. PO showed a notably smaller droplet size than PX (*p* < 0.05), which suggested that it possessed a more stable emulsion system. The stability of an emulsion significantly depends on its droplet size and size distribution. Emulsions with smaller droplets and lower PDI values are usually considered more stable [30,31,32]. Thongkaew et al. investigated the interactions between polyphenolic compounds (i.e., catechin and tannic acid) or plant infusions (i.e., grape seed and hibiscus) and a preheated whey protein isolate or its complex with pectin [33]. The authors found that polyphenols affect the particle size of whey protein above its isoelectric point. The mean size of whey protein increased with higher concentrations of the grape seed infusion and catechin, but decreased with the addition of hibiscus extract, and tannin. In this study, Aquasoya powder was dissolved in SPB, and the pH of the buffer (7.4) was higher than that of soy protein. Therefore, the phenolic compounds in PSE may have allowed interactions with the protein constituents in the rSBB powder, leading to the stabilization of emulsion droplets. The exact interactions between phenolic compounds in PSE and proteins from rSBB were not identified in this study. Previous research confirmed the interactions between RA and three whey proteins (β-lactoglobulin, α-lactalbumin, and bovine serum album) [34]. The hydrophobic effect was discovered to be the most dominant driving force in the binding of RA to these proteins. Meanwhile, the interactions between RA and soy proteins have yet been verified. However, such research is necessary to efficiently acquire a stable nanoemulsion containing PSE and Aquasoya from low-energy method, not only to improve bioaccessibility, but also to achieve a sustainable nanoemulsion formulated with minimum carbon emission.

The ζ-potential refers to the electrostatic potential at the electrical double layer surrounding a nanoparticle in solution. A higher ζ-potential suggests increased stability because of induced electrostatic repulsion between particles, which prevents aggregation [35]. In this study, PO possessed a lower ζ-potential than PX, which was not in accordance with the particle size analysis. The instability of PX might have contributed to this result. Ofir et al. compared the electroflocculation process of colloidal particles at different pH conditions (4, 6, 7.5, and 9) using ζ-potential and particle size as parameters and found that, throughout the flocculation time, the absolute ζ-potential value increased with increasing particle size [36]. The same phenomenon could have occurred in the PX. In other words, because the PX emulsion system was unstable, aggregation and flocculation could have caused PX to alleviate its instability by forming more stable and larger particles and increasing the electrostatic potential at the particle surface.

**Figure 4:**
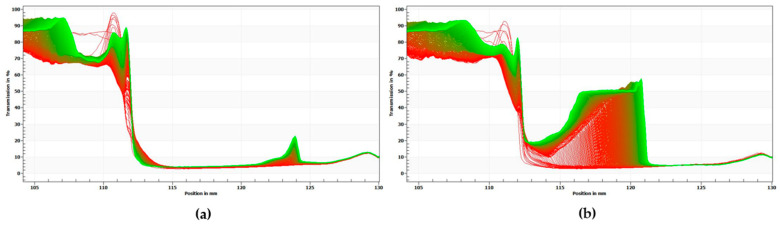
Transmission versus sample height profiles of emulsion samples. (**a**) PO, (**b**) PX. The red curves indicate the initial transmission profiles, whereas the green curves correspond to the final transmission profiles.

The results above are consistent with the results of centrifugal photosedimentometry analysis. Figure 4 shows the change in transmittance as a function of the height of the emulsion samples. The red curves indicate the initial transmission profiles and the green curves correspond to the final transmission profiles. As shown in Figure 3, the difference between the transmittance-height profiles was more distinct for PX than for PO, thus implying that PO possesses a more stable emulsion system.

There still remains a question about the stability of PO, which showed a stable emulsion system according to ζ-potential value, while the PDI values showed highly turbid and polydisperse droplets. This can be assumed to be derived from unsettling interactions between soy protein and perilla oil. In present research, the PX sample showed unstable emulsion system in present research. Also, Hu et al. prepared emulsions composed of perilla oil (2–4%) and soy protein isolate and formulated them with different homogenization intensities. As a result, the ζ-potential values of emulsion samples ranged from −8.48 ± 0.68 to −15.90 ± 1.04 mV, which represents highly unstable emulsion system [37]. Meanwhile, we formulated two emulsions with soybean oil as an oil phase, either of them containing PSE while the other one does not. They went through the photosedimentometry experiments and the results showed that the soybean oil emulsion without PSE showed the highest physical stability among four emulsion samples, including PO and PX. Combining these results, it can be assumed that soybean protein and perilla oil have conflicting nature, which unsettles the stability of emulsion system, thus causing fluctuation in particle size and higher PDI. But possibly the phenolic compounds from PSE effectively mediated this dispute between emulsion components, resulting in stable PO emulsion system. Furthermore, as Aquasoya powder contains numerous substances other than protein (carbohydrate, lipids and phenolic compounds), e.g., compared to soybean protein isolate, various interactions between components can be one of the other factors influencing the stability of emulsion.

**Figure 5:**
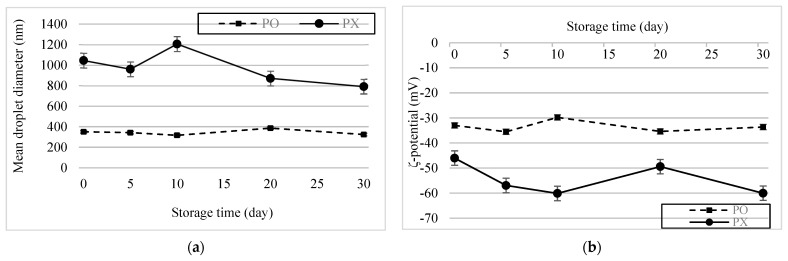
Storage stability of the emulsions. (**a**) Changes in particle size over 30 d. (**b**) ζ−potential change over 30 d.

The emulsion samples were stored for 30 d at 4 °C, after which changes in their physical characteristics were assessed (Figure 5a,b). No significant change in the particle size of PO was observed. During storage, the absolute potential of PX was greater than that of PO. However, it increased slightly after 5 d and then returned to its initial value after 20 d, whereas the PO remained constant (*p* < 0.05). This trend can be attributed to the instability of the PX emulsion system. As mentioned earlier, flocculation and aggregation cause changes in the ζ-potential of droplets. Therefore, interactions between labile emulsion droplets may have caused vacillations in the PX emulsion system. Based on these results, we concluded that PSE could stabilize O/W emulsions and help the system maintain its stability during storage.

### 3.4. Antioxidant Ability

The scavenging ability of DPPH and ABTS radicals is widely used to determine antioxidant ability because assays based on these stable radicals are easy to use, sensitive, and rapidly completed [38]. To assess whether the phenolic compounds in PSE retained their antioxidant capability after PO formulation and during storage, we conducted DPPH and ABTS assays for 30 d. Changes in the PV of the emulsions were also investigated to evaluate whether PSE prevents lipid oxidation.

**Figure 6:**
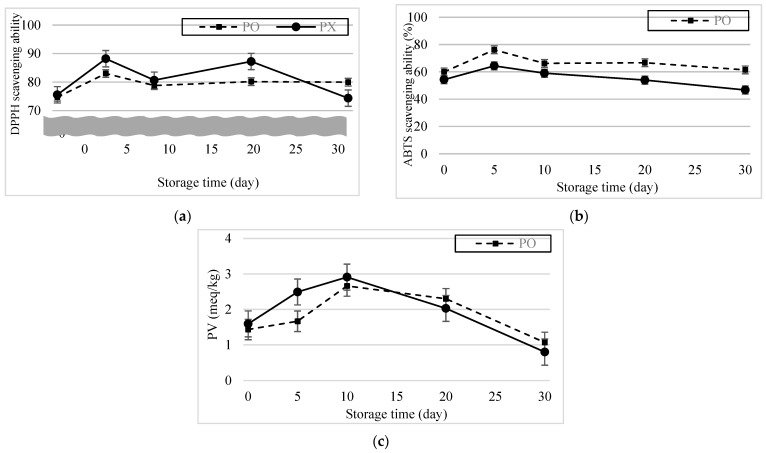
Antioxidant ability of the emulsions over 30 d. (**a**) DPPH radical scavenging ability. (**b**) ABTS radical scavenging ability. (**c**) Peroxide value change.

Figure 6a,b show the DPPH and ABTS radical scavenging ability results of the emulsions after 30 d. DPPH radical scavenging ability increased and was highest (PO, 83.02%; PX, 88.21%) 5 d after formation. And for the first 10 d, though the DPPH radical scavenging ability was lower in PO, there was no statistical difference between samples (*p* > 0.05). DPPH radical scavenging ability of PO was maintained for another 25 d, showing no statistical difference (*p* > 0.05). Meanwhile, DPPH radical scavenging ability of PX decreased to 74.37% and showed statistical difference (*p* < 0.05). The ABTS radical scavenging ability for both emulsions also increased dramatically and was highest 5 d after formation (PO, 76.20%; PX, 64.41%). Throughout the observation period, ABTS radical scavenging ability was higher in PO than in PX (*p* < 0.05). Differences between the results of the DPPH and ABTS assays may have resulted from the different reaction mechanisms of the radicals. According to Schaich et al. (2015), radicals can be quenched by either hydrogen atom transfer (HAT) or single-electron transfer (SET) [39]. Although both reactions form stable species from radicals, their kinetics and dependence on the system conditions are tremendously different. SET occurs very quickly and is not diffusion controlled but influenced by pH; by contrast, HAT is decelerated by diffusion and independent of the pH. The dominance of the scavenging mechanisms in ABTS and DPPH are also different. Tian and Schaich classified different antioxidant compounds into six groups according to the patterns of the ABTS reaction based on their initial and subsequent reaction rates and concentration dependence [40]. RA belongs to group 3, which shows a slow initial absorbance drop, followed by a continuous reaction thereafter, because the initial SET is hindered by bulky side groups and multiple rings, whereas the latter reaction is a result of HAT. DPPH reactions are mostly attributed to HAT. However, in some circumstances, when the reaction occurs in strong hydrogen-bonding solvents, the release of hydrogen is interrupted and SET becomes more dominant [40]. The DPPH reaction kinetics can also be categorized into four groups depending on the reaction rate, and RA is associated with group 3, which involves a moderate speed [41]. Thus, the different radical scavenging mechanisms in ABTS Scavenging ability and DPPH scavenging ability could lead to different scavenging rates and discrete consequences.

Phenolic compounds in the rSBB powder could also explain the difference in the results of the radical assays. Cooking water from SBB contains approximately 16 g of polyphenols per 100 g of CW [3]. He et al. performed nuclear magnetic resonance analysis of yellow soybean, black soybean, and SBB cooking water, and identified phenolic compounds such as resveratrol and glycitin among 20 investigated compounds [42]. Resveratrol is a naturally occurring phytoalexin that has effective radical scavenging ability and reducing power. Glycitin is known for its antioxidant, antiallergic, and anti-osteoporosis effects [43]. Because both reagents can potentially react with free radicals, they can compete with phenolic compounds from PSE. This supposition can be inferred from the ABTS and DPPH assay results for PSE. When PSE was dissolved in a solvent (ethanol:SPB, 1:3 *v*/*v*) at the same concentration as PO (0.4 mg/mL), the ABTS scavenging ability was 84.58% and DPPH scavenging ability was 35.83%. In other words, although the ABTS scavenging ability of PSE was significantly higher than that of the emulsion samples, the DPPH radical scavenging ability results were notably lower. Taking these findings together, we believe that various compositions of the phenolic compounds in the emulsion samples may have caused different kinetics in the experiment, resulting in different antioxidant capabilities.

Perilla oil is known to contain high contents of unsaturated fatty acids such as linolenic acid, linoleic acid and oleic acid, which contribute to their health-beneficial functions in humans [44,45]. However, because unsaturated fats are vulnerable to lipid oxidation, perilla oil easily deteriorates during storage [46], which limits its application in emulsions. Figure 6c shows the PV change of the emulsions after 30 d. The PV of PX was slightly higher than that of PO and changed more rapidly in the former than in the latter. Given the ABTS, DPPH assay, and PV results, we concluded that PO possesses and is able to maintain its antioxidant activity derived from the phenolic compounds in PSE.

## 4. Conclusions

In this study, a sustainable O/W emulsion containing PSE was formulated with rSBB and its physical stability and antioxidant were evaluated. The physical stability of the emulsion systems significantly improved in the PSE-incorporated emulsion and was maintained over 30 d of storage. DPPH and ABTS radical scavenging assays showed inconsistent results. The DPPH scavenging ability was slightly higher in PX (no statistical difference), whereas the ABTS scavenging ability was prominently higher in PO. However, as the change in PV was more gradual in PO than in PX, it was concluded that PO possesses antioxidant properties derived from the phenolic compounds in PSE. This results suggest that upcycled Aquasoya powder could be applied in formulating stable O/W emulsions containing bioactive compounds from perilla skin. Also, PSE may work as an effective antioxidant reagent, hindering lipid oxidation in the PSE-incorporated emulsion system. However, as the amount of extracted RA was lower in PSE compared to that from previous studies, further studies for optimal setting of RA extraction in perilla are required. Also, exact interactions between RA and rSBB should be identified for better understanding of the emulsion stability in present study, which can eventually lead to further studies about nanoemulsion containing perilla skin extract and upcycled Aquasoya powder with lower energy input. These deserve a more in-depth study, which is the goal of our next research on their practical application in food products.

## Figures and Tables

**Table 1:** RA contents in perilla skin and powder.

	RA Content in Perilla (mg/g)	RA Content in Extract Powder (mg/g)
Perilla skin	0.53 ± 0.12 ^b^	31.06 ± 2.57 ^b^
Perilla powder	1.21 ± 0.28 ^a^	34.82 ± 0.50 ^a^

RA, rosmarinic acid. Each experiment was performed thrice, and all values are expressed as mean ± standard deviation (*p* < 0.05). ^ab^ Means with different letters differ significantly according to the Mann–Whitney U test with *t*-test.

**Table 2:** Physical characteristics of the emulsion samples immediately after formation.

	Mean Droplet Diameter (nm)	PDI	ζ-Potential (mV)
PO	350.57 ± 9.60 ^b^	0.477 ± 0.09 ^N.S^	−32.99 ± 4.51 ^b^
PX	1045.37 ± 142.63 ^a^	0.613 ± 0.12 ^N.S^	−46.02 ± 4.48 ^a^

PDI, polydispersity index; PO, emulsion samples with perilla skin extract (PSE); PX, emulsion samples without PSE. Each experiment was performed thrice, and all values are expressed as mean ± standard deviation (*p* < 0.05). ^ab^ Means with different letters differ significantly according to the Mann–Whitney U test with *t*-test. ^N.S^ Not significant.

## Data Availability

The original contributions presented in the study are included in the article, further inquiries can be directed to the corresponding author.

## References

[1] Crippa M., Solazzo E., Guizzardi D., Monforti-Ferrario F., Tubiello F.N., Leip A. (2021). Food systems are responsible for a third of global anthropogenic GHG emissions. Nat. Food.

[2] Aschemann-Witzel J., Asioli D., Banovic M., Perito M.A., Peschel A.O., Stancu V. (2023). Defining upcycled food: The dual role of upcycling in reducing food loss and waste. Trends Food Sci. Technol..

[3] Echeverria-Jaramillo E., Kim Y.-H., Nam Y.-R., Zheng Y.-F., Cho J.Y., Hong W.S., Kang S.J., Kim J.H., Shim Y.Y., Shin W.-S. (2021). Revalorization of the cooking water (Aquafaba) from soybean varieties generated as a by-product of food manufacturing in Korea. Foods.

[4] Echeverria-Jaramillo E., Shin W.S. (2022). Effects of concentration methods on the characteristics of spray-dried black soybean cooking water. Int. J. Food Sci. Technol..

[5] Kim M.-J., Shin W.-S. (2022). Structural and functional modification of proteins from black soybean Aquasoya via ultrasonication. Ultrason. Sonochem..

[6] Kim Y.-H., Shin W.-S. (2022). Evaluation of the physicochemical and functional properties of aquasoya (Glycine max Merr.) powder for vegan muffin preparation. Foods.

[7] Hashimoto M., Matsuzaki K., Hossain S., Ito T., Wakatsuki H., Tanabe Y., Ohno M., Kato S., Yamashita K., Shido O. (2021). Perilla seed oil enhances cognitive function and mental health in healthy elderly Japanese individuals by enhancing the biological antioxidant potential. Foods.

[8] Yamamoto H., Ogawa T. (2002). Antimicrobial activity of perilla seed polyphenols against oral pathogenic bacteria. Biosci. Biotechnol. Biochem..

[9] Petersen M., Simmonds M.S. (2003). Rosmarinic acid. Phytochemistry.

[10] Lee J.H., Park K.H., Lee M.-H., Kim H.-T., Seo W.D., Kim J.Y., Baek I.-Y., Jang D.S., Ha T.J. (2013). Identification, characterisation, and quantification of phenolic compounds in the antioxidant activity-containing fraction from the seeds of Korean perilla (*Perilla frutescens*) cultivars. Food Chem..

[11] Gaihre Y.R., Iwamoto A., Oogai S., Hamajima H., Tsuge K., Nagata Y., Yanagita T. (2022). Perilla pomace obtained from four different varieties have different levels and types of polyphenols and anti-allergic activity. Cytotechnology.

[12] Hu N., Zhang K., Li Y., Hou T., Zhang Z., Li H. (2021). Glycine betaine enhanced foam separation for recovering and enriching protein from the crude extract of perilla seed meal. Sep. Purif. Technol..

[13] Hwang Y.J., Kim J.M., Yoon K.Y. (2020). Characteristics of water-soluble polysaccharides extracts produced from perilla seed meal via enzymatic hydrolysis. CyTA-J. Food.

[14] Zhu J., Fu Q. (2012). Optimization of ultrasound-assisted extraction process of perilla seed meal proteins. Food Sci. Biotechnol..

[15] Chumphukam O., Pintha K., Khanaree C., Chewonarin T., Chaiwangyen W., Tantipaiboonwong P., Suttajit M., Khantamat O. (2018). Potential anti-mutagenicity, antioxidant, and anti-inflammatory capacities of the extract from perilla seed meal. J. Food Biochem..

[16] Marchev A.S. (2021). Rosmarinic acid-From bench to valuable applications in food industry. Trends Food Sci. Technol..

[17] Guan Z., Li S., Lin Z., Yang R., Zhao Y., Liu J., Yang S., Chen A. (2014). Identification and quantitation of phenolic compounds from the seed and pomace of Perilla frutescens using HPLC/PDA and HPLC–ESI/QTOF/MS/MS. Phytochem. Anal..

[18] Hong E., Kim G.H. (2010). Comparison of extraction conditions for phenolic, flavonoid content and determination of rosmarinic acid from Perilla frutescens var. acuta. Int. J. Food Sci. Technol..

[19] Arranz E., Villalva M., Guri A., Ortego-Hernández E., Jaime L., Reglero G., Santoyo S., Corredig M. (2019). Protein matrices ensure safe and functional delivery of rosmarinic acid from marjoram (*Origanum majorana*) extracts. J. Sci. Food Agric..

[20] Han L., Lu K., Zhou S., Zhang S., Xie F., Qi B., Li Y. (2021). Development of an oil-in-water emulsion stabilized by a black bean protein-based nanocomplex for co-delivery of quercetin and perilla oil. LWT.

[21] Zhi Z., Yan L., Li H., Dewettinck K., Van der Meeren P., Liu R., Van Bockstaele F. (2022). A combined approach for modifying pea protein isolate to greatly improve its solubility and emulsifying stability. Food Chem..

[22] Huang K., Liu R., Zhang Y., Guan X. (2021). Characteristics of two cedarwood essential oil emulsions and their antioxidant and antibacterial activities. Food Chem..

[23] Homayoonfal M., Khodaiyan F., Mousavi M. (2015). Modelling and optimising of physicochemical features of walnut-oil beverage emulsions by implementation of response surface methodology: Effect of preparation conditions on emulsion stability. Food Chem..

[24] Korean National Institute of Crop Science Fostering of the Perilla Seeds, ‘Neulsaemi (Milyang No. 73)’, That Are Allele and Light in Seed Skin. https://nics.go.kr/bbs/view.do?m=100000126&bbsId=research&bbsSn=569607.

[25] Jacotet-Navarro M., Rombaut N., Fabiano-Tixier A.-S., Danguien M., Bily A., Chemat F. (2015). Ultrasound versus microwave as green processes for extraction of rosmarinic, carnosic and ursolic acids from rosemary. Ultrason. Sonochem..

[26] Ngo Y.L. (2018). Review on rosmarinic acid extraction, fractionation and its anti-diabetic potential. Food Chem. Toxicol..

[27] Dent M., Dragović-Uzelac V., Penić M., Bosiljkov T., Levaj B. (2013). The effect of extraction solvents, temperature and time on the composition and mass fraction of polyphenols in Dalmatian wild sage (*Salvia officinalis* L.) extracts. Food Technol. Biotechnol..

[28] Casanova F., Estevinho B., Santos L. (2016). Preliminary studies of rosmarinic acid microencapsulation with chitosan and modified chitosan for topical delivery. Powder Technol..

[29] Pan Y., Tikekar R.V., Wang M.S., Avena-Bustillos R.J., Nitin N. (2015). Effect of barrier properties of zein colloidal particles and oil-in-water emulsions on oxidative stability of encapsulated bioactive compounds. Food Hydrocoll..

[30] Damodaran S., Parkin K.L., Fennema O.R. (2007). Fennema’s Food Chemistry.

[31] Papadopoulou A., Frazier R.A. (2004). Characterization of protein–polyphenol interactions. Trends Food Sci. Technol..

[32] Zhang Y., Tan C., Abbas S., Eric K., Xia S., Zhang X. (2015). Modified SPI improves the emulsion properties and oxidative stability of fish oil microcapsules. Food Hydrocoll..

[33] Thongkaew C., Gibis M., Hinrichs J., Weiss J. (2014). Polyphenol interactions with whey protein isolate and whey protein isolate–pectin coacervates. Food Hydrocoll..

[34] Lu Y., Zhao R., Wang C., Zhang X., Wang C. (2022). Deciphering the non-covalent binding patterns of three whey proteins with rosmarinic acid by multi-spectroscopic, molecular docking and molecular dynamics simulation approaches. Food Hydrocoll..

[35] Selvamani V. (2019). Stability studies on nanomaterials used in drugs. Characterization and Biology of Nanomaterials for Drug Delivery.

[36] Ofir E., Oren Y., Adin A. (2007). Electroflocculation: The effect of zeta-potential on particle size. Desalination.

[37] Hu M., Xie F., Zhang S., Li Y., Qi B. (2020). Homogenization pressure and soybean protein concentration impact the stability of perilla oil nanoemulsions. Food Hydrocoll..

[38] Olszowy M., Dawidowicz A.L. (2018). Is it possible to use the DPPH and ABTS methods for reliable estimation of antioxidant power of colored compounds?. Chem. Pap..

[39] Schaich K., Tian X., Xie J. (2015). Hurdles and pitfalls in measuring antioxidant efficacy: A critical evaluation of ABTS, DPPH, and ORAC assays. J. Funct. Foods.

[40] Tian X., Schaich K. (2013). Effects of molecular structure on kinetics and dynamics of the trolox equivalent antioxidant capacity assay with ABTS+•. J. Agric. Food Chem..

[41] Xie J., Schaich K. (2014). Re-evaluation of the 2, 2-diphenyl-1-picrylhydrazyl free radical (DPPH) assay for antioxidant activity. J. Agric. Food Chem..

[42] He Y., Shim Y.Y., Shen J., Kim J.H., Cho J.Y., Hong W.S., Meda V., Reaney M.J. (2021). Aquafaba from Korean Soybean II: Physicochemical properties and composition characterized by NMR analysis. Foods.

[43] Gülçin İ. (2010). Antioxidant properties of resveratrol: A structure–activity insight. Innov. Food Sci. Emerg. Technol..

[44] Kim Y.M., Huh J.S., Lim Y., Cho M. (2015). Soy Isoflavone Glycitin (4′-Hydroxy-6-Methoxyisoflavone-7-D-Glucoside) Promotes Human Dermal Fibroblast Cell Proliferation and Migration via TGF-β Signaling. Phytother. Res..

[45] Choi H.J., Song B.R., Kim J.E., Bae S.J., Choi Y.J., Lee S.J., Gong J.E., Lee H.S., Lee C.Y., Kim B.-H. (2020). Therapeutic effects of cold-pressed perilla oil mainly consisting of linolenic acid, oleic acid and linoleic acid on UV-induced photoaging in NHDF cells and SKH-1 hairless mice. Molecules.

[46] Shim S.D., Lee S.J. (2011). Shelf-life prediction of perilla oil by considering the induction period of lipid oxidation. Eur. J. Lipid Sci. Technol..

